# Evaluation of Molecularly Imprinted Polymers for Point-of-Care Testing for Cardiovascular Disease

**DOI:** 10.3390/s19163485

**Published:** 2019-08-09

**Authors:** Brian Regan, Fiona Boyle, Richard O’Kennedy, David Collins

**Affiliations:** 1 School of Biotechnology, Dublin City University, Dublin 9, Ireland; 2 Research Complex, Hamad Bin Khalifa University, Qatar Foundation, Doha, Qatar

**Keywords:** point-of-care, cardiovascular disease, biomimetic sensors, molecularly imprinted polymers, cardiac biomarker, biomarker detection

## Abstract

Molecular imprinting is a rapidly growing area of interest involving the synthesis of artificial recognition elements that enable the separation of analyte from a sample matrix and its determination. Traditionally, this approach can be successfully applied to small analyte (<1.5 kDa) separation/ extraction, but, more recently it is finding utility in biomimetic sensors. These sensors consist of a recognition element and a transducer similar to their biosensor counterparts, however, the fundamental distinction is that biomimetic sensors employ an artificial recognition element. Molecularly imprinted polymers (MIPs) employed as the recognition elements in biomimetic sensors contain binding sites complementary in shape and functionality to their target analyte. Despite the growing interest in molecularly imprinting techniques, the commercial adoption of this technology is yet to be widely realised for blood sample analysis. This review aims to assess the applicability of this technology for the point-of-care testing (POCT) of cardiovascular disease-related biomarkers. More specifically, molecular imprinting is critically evaluated with respect to the detection of cardiac biomarkers indicative of acute coronary syndrome (ACS), such as the cardiac troponins (cTns). The challenges associated with the synthesis of MIPs for protein detection are outlined, in addition to enhancement techniques that ultimately improve the analytical performance of biomimetic sensors. The mechanism of detection employed to convert the analyte concentration into a measurable signal in biomimetic sensors will be discussed. Furthermore, the analytical performance of these sensors will be compared with biosensors and their potential implementation within clinical settings will be considered. In addition, the most suitable application of these sensors for cardiovascular assessment will be presented.

## 1. Introduction

**Figure 1:**
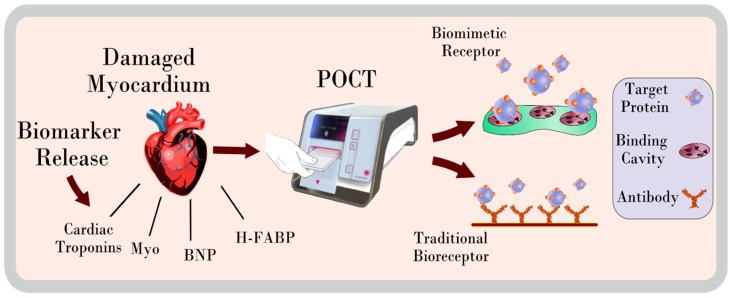
Point-of-care testing (POCT) utilises traditional bioreceptors, such as antibodies, for cardiac biomarker measurements. Traditional bioreceptors are integral in the detection of released cardiac biomarkers for POCT. Myo—Myoglobin, BNP—Brain natriuretic peptide, H-FABP—Heart type fatty acid binding protein.

Early detection and intervention of cardiovascular disease (CVD) continues to be a significant challenge for clinicians and healthcare providers worldwide. It is responsible for a vast number of deaths annually and often represents the most significant healthcare expenditure of all disease categories [1,2,3]. Typically, a broad range of techniques and resources are employed during the process of diagnosing and managing CVD. Crucially, the ability to effectively and rapidly determine the occurrence and subsequent severity of CVD enables highly informed and effective decision making and thus the most suitable course of action. Point-of-care testing (POCT) enables rapid assessment, offering an alternative to traditional methods and, in doing so, provides the opportunity to decentralise various diagnostic resources [4,5]. Electing to implement POCT can significantly increase the diagnostic capacity and efficiency of an assessment procedure determining the ‘rule-in/rule-out’ of acute myocardial infarction (AMI) and other related conditions [6,7]. The justification of POCT for cardiac biomarkers is further supported by the often unclear and vague nature of several symptoms attributed to CVD, chiefly the onset of chest pain, which itself represents 4.7% of all emergency department visits in America and results in a discharge rate of 57%, of which, 90% were diagnosed as non-specific chest pain [8]. This further emphasises the need to optimise the efficiency of the triaging process for which POCT can play a highly influential role. Additionally, the implementation of POCT in the Emergency Department (ED) was shown to reduce both the turnaround time and period between testing and administering the required cardiac treatment when compared to central laboratory testing. In addition, it can further reduce the length of stay in an ED by up to 27% with successful implementation of cardiac troponin (cTn) measurements [9,10]. Despite the cTns being regarded as the gold standard cardiac biomarkers and used as the principal indicators of AMI, the sensitivity of detection achievable using POCT has generally been inferior to that of central laboratory assays [11,12,13]. However, many studies have indicated that both approaches demonstrate comparable characteristics, with several recent findings suggesting the latest point-of-care (POC) platforms display comparable analytical performance to laboratory-scale high-sensitivity cTn (hs-cTn) assays. An example of a POC platform and recognition elements that can be used to capture target analyte are depicted in Figure 1 [14,15,16,17,18]. Point-of-care testing can be implemented in various configurations, facilitating detection of multiple biomarkers simultaneously in a multiplexed format, in conjunction with a devised algorithm to optimise the diagnostic capacity of the platform. Point-of-care testing can be performed in a wide variety of test settings, enabling direct cardiac assessment outside of the hospital and in limited-resource settings, albeit, the specific POC platform may vary depending on individual performance and setting requirements. These requirements are key drivers in the ongoing development of novel diagnostic platforms, incorporating innovative techniques that expand the potential application and effectiveness of POCT.

Isolating analytes from complex test samples is a fundamental feature of integrated sensors that are central to the operation of rapid diagnostic devices. Traditionally, high affinity biorecognition elements have been key to the development of these platforms, enabling the separation, and facilitating the detection of biomarkers. However, recently, imprinting techniques have been proposed as an alternative for the fabrication of chemically defined recognition elements. Molecular imprinting involves generating synthetic receptors featuring adapted cavities that facilitate highly specific binding of target analytes. Typically, this process consists of polymerising functional monomers in the presence of template molecules and introducing cross-linkers, in an appropriate porogenic solvent, to retain the specific recognition structure prior to template removal. In relation to established imprinting techniques, several variations exist that are often more suited to certain templates and particular end applications of molecularly imprinted polymers (MIPs). However, difficulties can occur when using biomolecules as templates (proteins being particularly problematic) due to their high sensitivity to many solvents which are often employed in the imprinting process, resulting in the denaturing of the template molecule. Traditionally, MIPs have been synthesised successfully for smaller molecules (<1.5 kDa), however, complications often arise regarding macromolecule templates and the subsequent ability to effectively bind to the analyte [19,20]. This aspect has historically rendered MIPs as a less appealing alternative for consideration in cardiac disease detection-based POC platforms since cardiac biomarkers are susceptible to degradation in suboptimal environments (the majority of cardiac biomarkers also being larger than 1.5 kDa) [21,22]. Moreover, the efficacy of a MIP is greatly influenced by the specific technique employed during synthesis, with a variety of methods reported, many of whom are extensively reviewed throughout the literature [19,23,24,25]. Some notable applications of MIPs include trace analyte extraction, chromatographic separation and use in various chemical sensors, although, they have yet to find widespread commercial use as a solid phase [26,27,28,29,30,31]. However, MIP-based biomimetic sensors in many ways can emulate conventional biosensors, although there are some key distinctions that heavily dictate their configuration. For example, the innate structure of MIPs mean that MIP-based biomimetic sensors often require several progressive (and sometimes complex) synthesis steps, and in many cases the format of the MIP may dictate the specific nature of detection technique which can be used. Furthermore, various synthesis enhancement techniques support the production of MIPs with improved separation capabilities and decreased batch variability, thus advancing their potential application to POC platforms.

This review aims to assess the use of imprinting techniques for the generation of biomimetic sensors targeting cardiac biomarkers and, subsequently, an evaluation of the potential application of these sensors for POCT. The inherent issues associated with protein imprinted polymers (PIPs) will be outlined in detail, in addition to examining the challenges of using protein templates and the characteristics that the derived sensors display. These important aspects influence the potential applicability of this technology for POCT as there is a requirement to adhere to a pre-defined set of criteria for a diagnostic platform to be considered compatible with POC standards. The analytical performance of the biomimetic sensors will be compared against developed biosensors targeting cardiac biomarkers. Additionally, the mechanism of detection employed in some of the cardiac biomimetic sensors will be illustrated, highlighting how these approaches diverge from their biosensor counterparts. Furthermore, the practical challenges of incorporating biomimetic sensors into cardiovascular assessment in clinical settings and the potential contribution of MIPs within POCT for CVD are discussed.

## 2. Protein Detection

Traditional biosensors utilise biorecognition elements such as antibodies, enzymes, cells, and aptamers (amongst others) to capture and measure the targeted analyte. The innate qualities that these biorecognition elements display facilitates the fabrication of numerous biosensors with high specificity and sensitivity for various cardiac biomarkers [32,33]. Consequently, these are key to the development of reliable POC platforms for cardiac assessment, providing the foundation for highly automated sample analysis. Point-of-care testing is a diagnostic technique that relies upon a high level of automation, encapsulating multiple processes within a practical device. Numerous definitions of POCT have been proposed with Luppa et al. suggesting there are nine typical characteristics of POCT [34,35,36,37], although they identify three essential aspects, i.e., conducting the test in the patient’s proximity while providing rapid results that can, in turn, enable rapid diagnosis. These are fundamental characteristics of POCT in which the addition of MIPs should not only preserve, but enhance its applicability. Moreover, detecting the most widely assessed cardiac biomarkers, the cTns, have several reported challenges regarding obtaining accurate measurements in serum [38,39,40,41,42,43,44,45,46,47]. These difficulties are attributed to an array of factors, ranging from interfering molecules, the presence of skeletal muscle troponin isoforms, a lack of standardisation and a distinct 99th percentile upper reference limit (URL) disparity between genders, amongst others. The occurrence of these interferences and the challenges in the detection of cTn highlights the potential performance limitations of POCT for single cardiac biomarker analysis in time-critical settings, and presents itself as an additional hindrance for the incorporation of PIPs into these devices.

### 2.1. Challenges of Protein Detection Using PIPs

Molecularly imprinted polymers have been shown to perform well in several applications that target small molecules, often exceeding conventional methods and providing a more cost-effective approach [48,49,50]. However, targeting macromolecules such as proteins introduces additional challenges, particularly affecting the synthesis of PIPs and the analytical performance of the subsequent biomimetic sensor. Several key challenges are outlined in Table 1, in addition to their significance in relation to the performance of the fabricated sensor.

Typically, these issues are more prevalent for MIPs synthesised from bulk imprinting. Thus alternative techniques, such as surface imprinting and epitope imprinting are often preferred for larger biomolecules like proteins. Furthermore, various methods have been proposed to mitigate these challenges and improve the performance of PIPs, several of which will be outlined throughout this section with further solutions previously detailed by Chen et al. [51].

### 2.2. Heterogeneity

The occurrence of heterogeneous binding sites is a major deficiency present in PIPs. In fact, these are present in the majority of MIPs and are dependent upon the monomer and solvent selection, imprinting conditions, template removal and post-processing of the material [52]. However, this is exacerbated for PIPs by the size, conformational flexibility and the complex functional nature of proteins. Studies have shown that heterogeneity reduces both binding site specificity and the affinity towards the protein, as well as impacting the binding kinetics [52,53]. Promising methods aiming to address this issue involve immobilising the protein on a solid substrate, particularly utilising nanomaterials to ensure the proteins are arranged in a specified orientation before polymerisation [54,55,56,57]. Moreira et al. covalently attached cardiac troponin T (cTnT) to multiwalled carbon nanotubes (MWCNTs) to increase the abundance of homogenous binding sites [58]. The authors employed EDC/NHS coupling to form a stable amide bond between the MWCNTs and cTnT before introducing the pre-polymerisation mixture, although this approach does not guarantee uniform orientation. The polymerisation was performed at room temperature in a physiological buffer to maintain the original structure of the protein template. Another biomimetic sensor constructed by the same group targeted myoglobin (Myo), a globular hemeprotein found in the myocardium and skeletal muscle that provides value when ruling out AMI [59,60,61]. A self-assembled monolayer (SAM) consisting of the aminothiol cysteamine and the homobifunctional cross-linker glutaraldehyde was deposited upon the gold working electrode to facilitate the covalent attachment of the Myo template [62]. Atomic force microscopic analysis revealed the cavities to be in the range of 10 nm in size, suggesting that the Myo was imprinted in a dimeric form, as in its normal monomeric state it measures approximately 3.5 nm. Nonetheless, the PIP could selectively bind to Myo in the presence of several interfering molecules, thus facilitating accurate measurements below the clinical ‘cut-off’ concentration, i.e., the pre-defined biomarker concentration that, if exceeded, indicates adverse cardiac health and can lead to a diagnosis of AMI [63].

In addition to restricting the biomimetic sensor performance, heterogeneous cavities can impede the complete removal of the template molecule, therefore compromising the performance of the sensor due to the occupancy of high-affinity binding sites. Furthermore, heterogeneous binding sites can limit the complete removal of the template protein and in turn, lead to template leaching during the sensing process. Enzymatic digestion is an alternative approach that enables template removal under mild conditions, with proteinase K having been shown to remove the majority of the template from high-affinity binding sites [64,65]. This method was incorporated in the design of a biomimetic sensor targeting Myo, cleaving the template protein into small amino acid residues prior to washing [66], however, it was reported that enzymatic digestion alone often does not ensure complete template removal [64,67]. This is demonstrated by Venton and Gudipati when they assessed the retention of Myo within a bulk prepared PIP following pronase digestion [68]. In this work, the Myo was removed quite rapidly (10%/h) in the first 2 h, however after 160 h only 35% of the protein was removed, leaving whole/ partial template molecules within the majority of the binding sites. Another study illustrated improved template extraction by denaturing the template protein using a surfactant and subsequent reduction of the disulfide bonds [67]. Ribeiro et al. utilised trypsin to enzymatically digest Myo and evaluated the template extraction efficiency compared to washing the imprinted polymer layer overnight with a solution of PBS, methanol and sodium dodecyl sulphate (SDS) [69]. In this work, the enzymatic digestion resulted in incomplete template removal, with the authors suggesting that protein fragments remained in the binding sites and rendered these cavities inaccessible. In contrast, the washing protocol removed the majority of the template protein and enabled rebinding of Myo for subsequent measurements.

### 2.3. Binding Kinetics

In comparison to bulk imprinting (an approach that generates ‘3D imprints’ in which binding sites are located throughout the imprinted structure) studies have shown that equilibrium can be achieved up to 18 times faster for MIPs synthesised using surface imprinting [70]. Surface imprinted polymers consist of binding sites that are formed on the surface of a substrate and are more accessible than those produced by bulk imprinting, although typically, these MIPs contain less cavities, thus limiting their overall binding capacity [71]. Other strategies have also proven effective at producing thin layer MIPs with favorable binding kinetics, such as electropolymerisation—in this case a potential is applied to an electrode enabling sufficient control over the polymer layer thickness [72,73]. This imprinting technique was implemented in the fabrication of a biomimetic sensor targeting Myo with the electropolymerisation of *o*-phenylenediamine (PDA, 1,2-diaminobenzene) being controlled by cyclic voltammetry [74]. Here, the authors performed square wave voltammetry measurements following a 10-min incubation period, detecting Myo based solely on the reduction of the protein at concentrations below the clinical ‘cut-off’. Another study assessed analyte mass transfer for PIPs and determined that protein diffusion for surface imprinting was up to 30 times faster in contrast to bulk imprinting [75]. This particular study synthesised the PIPs using hierarchal imprinting, a form of surface imprinting that involves attaching the template molecule to a pore-defining surrogate to ensure the imprinted polymer retains structural porosity after polymerisation [76]. Consequently, this biomimetic receptor was capable of reaching equilibrium for human albumin serum in 20 min. Interestingly, the authors used porcine serum albumin as a dummy template, a structural analogue to human serum albumin—due to its widespread availability—which would permit mass production of the PIPs. Dummy templates have also been shown to be effective at reducing the impact that template leaching has on analyte detection. Depending on the biomimetic sensor configuration, their release during analyte measurement will prevent a corresponding response, although, it has been shown that dummy PIPs can suffer from poor specificity [51,77].

Analogues can be utilised to enhance other aspects of an imprinted polymer beyond simply mitigating leaching effects. This is demonstrated by another group that produced thin-film PIPs to target C-reactive protein (CRP) [78]. Several studies have highlighted the correlation between increased serum levels of CRP and a greater risk of adverse cardiac health, with some reporting its added prognostic value, particularly when measured in conjunction with several other biomarkers [79,80,81,82]. This work exploited the affinity of CRP towards its natural ligand, phosphocholine, to produce a highly specific biomimetic receptor capable of detecting clinically relevant concentrations of CRP in the presence of serum proteins. In this instance, the functional monomer employed by the authors was O-(4-nitrophenylphosphoryl)choline, a phosphorylcholine (PC) derivative which is an analogue of phosphocholine. This ready-made monomer is one of a wide range of available PC derivatives that can serve as suitable receptors for CRP among being key elements within various biomedical devices [83,84,85,86]. 

### 2.4. Solvent Compatability

A limiting feature of PIPs is the inability to perform their synthesis in many organic solvents due to the surface properties of template proteins, thus restricting the selection of compatible functional monomers [87,88]. The amino acid residues in proteins are affected by the properties of organic solvents and in turn, their structural arrangement is disturbed in non-aqueous solutions (although to a lesser extent in aprotic solvents) resulting in PIPs with binding cavities of low affinity [89,90]. Moreover, the challenge of selecting the most appropriate monomer for a specific protein template extends beyond the dilemma dictated by solvent compatibility. Hence, there are several factors that significantly influence the generation of specific binding sites. A study by Sullivan et al. demonstrated that acrylamide-based monomers that were smaller in size and that formed comparatively less hydrogen bonds with the side chains than the protein backbone, resulted in less secondary structural changes within the protein template prior to polymerisation [91]. Notably, if the conformational structure of the template protein is disturbed before or during polymerisation, the PIP will consist of binding sites that will inhibit rebinding. Additionally, other studies confirmed that monomer concentration is another considerable aspect that affects the quality of the binding sites, with excessive concentrations inducing unwanted interactions with the protein backbone [92,93]. These factors, among others, govern the characteristics of binding sites within PIPs and influence the shape and functional features of these cavities. Whitcombe et al. provide an extensive description of suitable monomers and cross-linkers utilised in the synthesis of PIPs and details several factors that influence their selection and how they interact with protein templates [94].

Epitope imprinting is a technique that displays promising attributes, in theory enabling the use of conventional monomers and cross-linkers due to the ease of template removal, insignificant conformational changes and reduced degradation [95,96]. This method relies upon targeting an epitope (traditionally a short amino acid sequence that is recognised by an antibody) as the area against which the PIP is produced to bind, to adequately capture the protein of interest. Bossi et al. developed PIPs through the implementation of the epitope approach for the recognition of N-terminal pro-B-type natriuretic peptide (NT-proBNP), an important biomarker of CVD [97]. Studies have shown that NT-proBNP is an excellent predictor of cardiovascular mortality, in addition to providing diagnostic sensitivity for AMI. However, a significant aspect of NT-proBNP measurement is the low clinical ‘cut-off’ concentration of 300 ng/L that must be detected [98,99,100,101]. The group developing PIPs for NT-proBNP conducted fingerprint analysis and identified the importance of selectively targeting certain epitopes to obtain ideal templates that ensure the binding capacity of the PIP was optimised. Online protein databases allowed the establishment of numerous potential polypeptide sequences present in NT-proBNP to be established based on specified criteria. Suitable PIP target epitopes were chosen by screening the sequences against a protein databank and assigning a probability of uniqueness score, i.e., the e-value generated by BLAST. Thus, PIPs of varying composition were synthesised for two separate polypeptide sequences and the performance characteristics of each were assessed. Noticeable differences between binding capacities and the imprinting factors for each were demonstrated. The imprinting factor is a widely used assessment standard relating to the binding ability of a MIP and is simply the ratio of bound analyte between the MIP and the control polymer (NIP) (NIP—synthesised by the same process as the MIP, but excludes the introduction of the template). The authors ultimately recommended a single PIP based on its ability to selectively bind to polypeptide fragments of cleaved NT-proBNP within 30 min. Although this is a refined and systematic process involving the identification of distinctive amino acid sequences, the dependence upon enzymatic digestion—prior to the rebinding of the cleaved epitopes—restricts its application in biomimetic sensors aimed at time critical analysis. Furthermore, this work highlighted the considerable impact that slight variations in the composition of the pre-polymerisation mixture and the monomer and cross-linker concentrations have on the performance of PIPs.

## 3. Detection Techniques

Central to the feasibility of POCT is the capacity to provide rapid analysis in the absence of sophisticated instrumentation. This requirement may somewhat discount the suitability of various techniques that have associated instruments that are bulky, costly or challenging to operate. Labelled biorecognition elements are common features of many biosensors and assays, and require a multi-step process that culminates in the formation of a sandwich complex, thus enabling precise analyte measurements.

### 3.1. Sandwich Complexes

**Figure 2:**
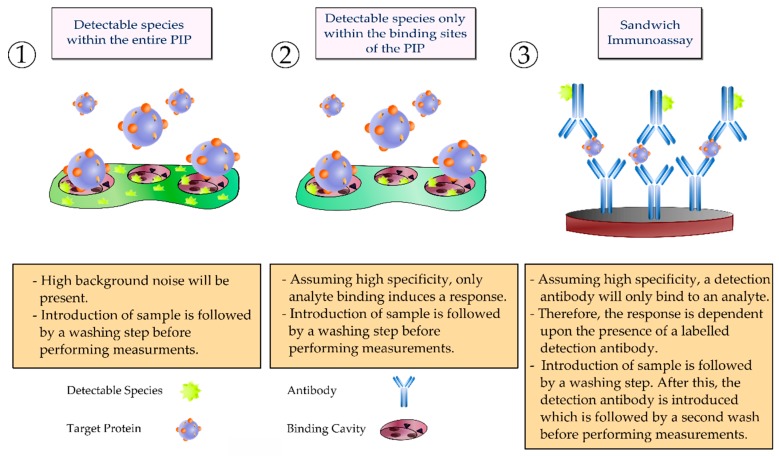
Alternative strategies for incorporating a detectable species within a protein imprinted polymer in comparison to an antibody sandwich complex for rapid and sensitive detection of an analyte.

In recent times, the vast majority of reported biosensors targeting cTnI employ electrochemical or luminescent-based detection techniques, with a large proportion of luminescent-based biosensors relying on secondary labelled antibodies [102]. Moreover, many commercially available POC platforms for the detection of cTnI also rely on the formation of sandwich complexes, with fluorescence detection being one of the most common techniques employed—similar to that depicted in Figure 2 [103]. This format is typically quite difficult to accomplish using PIPs, although the adoption of nanomaterials has enabled the fabrication of biomimetic sandwich complexes. The composition of biomimetic receptors often influences the selection of a suitable transducer mechanism for translating the analyte concentration, essentially dictating biomimetic sensor configuration. Furthermore, selecting an appropriate transducer mechanism is considered a critical step in the development of a biomimetic sensor and one that influences the utility of the MIP [104].

Some studies have reported the construction of sandwich complexes for protein detection, incorporating nanoparticles (NPs) and quantum dots (QDs) coated in an imprinted polymer layer, with others constructing compounds consisting of PIPs and secondary antibodies [105,106,107,108]. Although there is an apparent lack of reported sandwich techniques aimed at cardiac biomimetic sensing, Kim et al. have developed a CRP biomimetic assay using a PC analogue as the monomer, and horseradish peroxidase (HRP) labelled secondary antibody [109]. This assay could detect sufficient levels of CRP in diluted serum using absorbance measurements at 450 nm. Despite the period of incubation for the detection antibody not being disclosed, this assay would be unsuitable for rapid analysis as the initial CRP incubation period alone was 30 min.

**Figure 3:**
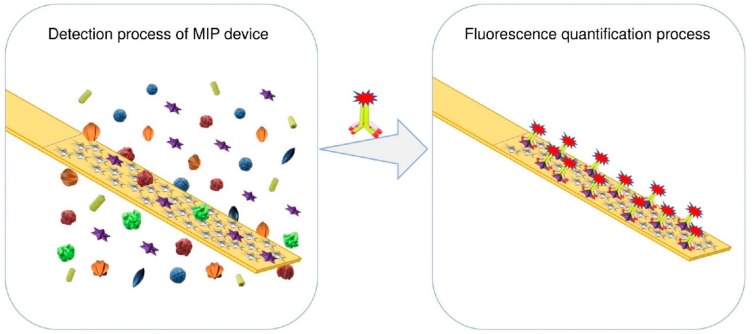
Detection scheme for a hybrid IL-1β biomimetic sensor [110] (Figure was re-printed with permission from the publisher).

Deng et al. presented the development of a biomimetic sensor targeting Interleukin-1β (IL-1β), a cytokine protein shown to be heavily implicated in the progression of CVD and that has been suggested as a prognostic biomarker of heart failure (although its value as a useful cardiac biomarker is often disputed, primarily due to its presence in extremely low concentrations) [110,111,112,113,114,115,116]. The authors performed the surface imprinting on a stainless steel ribbon, alluding to advantages associated with its mechanical properties compared to glass or ceramics. The PIP synthesis largely relied on the electrostatic potential between polydopamine, poly(ethyleneimine) and the template protein to generate the cavities after immersion in a dopamine solution. The detection of IL-1β was accomplished through the introduction of fluorescently labelled secondary antibodies conjugated to polystyrene beads that bind to the target protein, an illustration of which is depicted in Figure 3. This hybrid biomimetic sensor displayed a linear range of 25–400 ng/L and had a 10.2 ng/L limit of detection (LOD), demonstrating its capacity to detect IL-1β below a threshold of 49.1 ng/L that was recently identified as diagnostically relevant for CVD assessment [111].

### 3.2. Stimuli-Responsive Species

Synthesising MIPs from stimuli-responsive materials is a unique aspect that enables the production of multifunctional biomimetic receptors that can simplify the sensing process. Numerous studies report on a broad range of stimuli-responsive polymers and gels that respond to heat, light, pH, the presence of an electric field and specific biomolecules [25,117,118,119,120]. The use of fluorescent reporter molecules containing a functional group that can is possibly the most common approach to integrate a detectable species within a MIP [121] although MIPs synthesised in this manner often suffer from a high fluorescence background signal [115]. Hence, Sunayama et al. designed a monomer with specific functional groups to ensure that they were able to introduce a fluorescent reporter to the binding sites post-polymerisation [122]. Interestingly, rebinding of the template molecule to the binding sites increased the fluorescence intensity, with the authors also demonstrating that non-specific adsorption of molecules to the imprinted layer did not induce a fluorescence response. There is a lack of studies detailing the use of stimuli responsive MIPs targeting cardiac biomarkers, however, Turan et al. exploited temperature-responsive hydrogels for the detection of Myo [123]. Hydrogels are 3-dimensional polymer networks consisting of a hydrophilic structure that accommodates water absorption and are known to express anti-fouling properties, in addition to being ideal materials for incorporating stimuli-responsive features [124,125,126]. In this work the authors synthesised the gel from N-isopropylacrylamide (NIPA), a thermosensitive monomer, and 2-acrylamido-2-methyl-propanosulfonic acid (AMPS) at various temperatures in the region of the lower critical solution temperature and subsequently assessed the hydrogel performance. This work demonstrated the importance of the imprinting temperature on the structure of a hydrogel, with a considerable variation in pore size, selectivity, and Myo adsorption observed. Crucially, the binding kinetics displayed by the synthesised hydrogels are not favorable for POC integration. Other studies have described the synthesis of supermacroporous cryogels for the extraction of Myo [127,128]. These gels are synthesised below 0 °C and exhibit superior mechanical properties in comparison to standard hydrogels, in addition to an increased porous structure [129]. The use of gel-based extraction columns facilitated protein enrichment by removing high-abundance proteins from complex samples, which is often required before assessing serum for low-abundance biomarkers [130,131]. The enrichment of Myo, however, is not a priority for POCT due to its relative ease of detection, although this approach could be valuable for other biomolecules that are in the circulation in minute concentrations and where the availability of highly specific antibodies against them is very limited.

### 3.3. Signal Quenching

**Figure 4:**
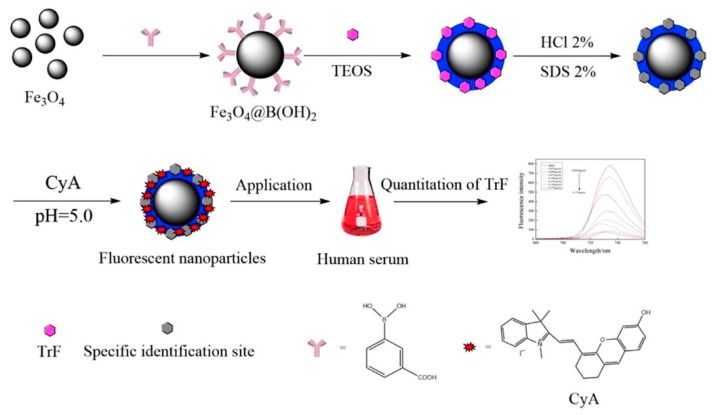
Detection scheme for protein imprinted polymer magnetic fluorescent NPs for transferrin measurements [132]; Fe_3_O_4_—Iron oxide, Fe_3_O_4_@(B(OH)_2_—Boronic acid-modified Fe_3_O_4_, TEOS—Ethylsilicate, HCl—Hydrochloric acid, CyA—Semi-cyanine fluorescent compound, TrF—Transferrin (Figure was re-printed with permission from the publisher).

‘Label-free’ detection is often considered an attractive approach suited to POCT due to the reduced complexity of the sensing process. Zhang et al. have reported on the development of PIP magnetic fluorescent NPs for the detection of transferrin, as illustrated in Figure 4 [132]. Although not a specific cardiac biomarker, this iron-binding glycoprotein was shown to provide a relatively high level of predictive value in assessing cardiovascular mortality when the serum iron content is assessed against the total available transferrin in circulation [133,134,135]. In this work, the authors exploited the affinity of glycoproteins towards boronic acid in the synthesis of the molecularly imprinted Fe_3_O_4_ NPs. After forming the binding sites, electrostatic adsorption of a semi-cyanine fluorescent compound onto the PIP provided the mechanism for detection. Transferrin measurements were determined from the quenching effects that occur due to photoinduced electron transfer between the transferrin and the fluorophore which are influenced by the net charge of transferrin at neutral pH. Therefore, the fluorescence signal becomes increasingly attenuated for higher sample concentrations of transferrin.

A similar approach was reported for the detection of Myo, in which the authors described a signal quenching technique utilising fluorescent QDs and involved a performance characteristic comparison study between bulk and surface imprinted polymers for the detection of Myo [136]. Quantum dots are semiconductor NPs that display unique luminescent properties and have recently been adopted for the development of various sensors among finding utility in other applications [137,138]. Re-binding of Myo masked the QDs within the MIPs which reduced the fluorescence signal intensity. The authors experimentally determined that the surface imprinted PIP-QDs were capable of detecting lower concentrations of Myo and had a wider linear detection range than the bulk imprinted polymers. Myoglobin measurements were conducted following 5 and 30-min incubation periods, with a LOD of 1.47 ng/L after the 5-min incubation period. Moreover, the authors allude to the improved sensitivity following a 30-min incubation period which produced an impressive LOD of 135.9 pg/L. However, a notable limitation of the study is the complete quenching of the fluorescence signal for Myo concentrations below reported ‘cut-off’ thresholds, which is particularly evident for the surface imprinted PIP-QDs [63,139]. Hence, further development would be necessary to detect higher Myo concentrations and for this QD-based detection technique to be clinically suitable.

**Figure 5:**
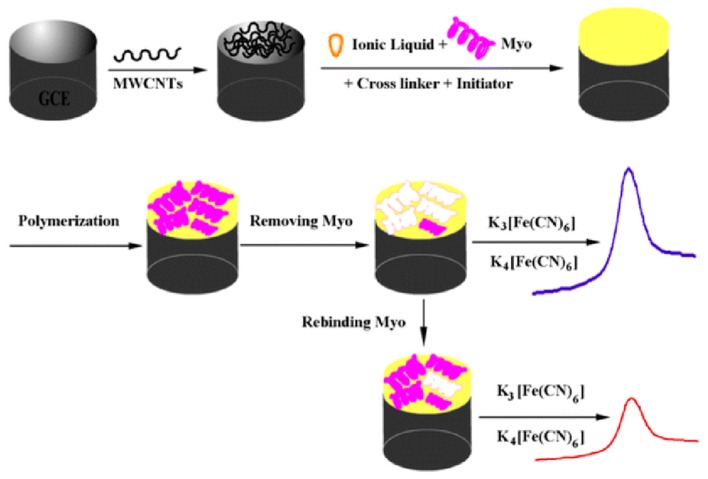
Electropolymerisation of protein imprinted polymer from ionic liquid onto a working electrode and the detection of myoglobin [140] (reprinted with permission from the publisher). K3[Fe(CN)6]—Potassium Ferricyanide, K4[Fe(CN)6]—Potassium Ferrocyanide.

A slightly modified approach to the signal quenching methods presented above was demonstrated in the development of an electrochemical-based biomimetic sensor targeting Myo [140]. A PIP was synthesised using an ionic liquid monomer to overcome common challenges that are regularly encountered, such as performing the polymer synthesis in an aqueous solution. Ionic liquids are salts that are liquid at room temperature and have most recently been considered as functional building blocks in polymer structures [141]. Moreover, they display favorable properties such as; high thermal and electrochemical stability, mechanical durability and high ionic conductivity [141,142]. In this work, MWCNTs were deposited on the surface of the glassy carbon electrode (GCE) to improve the performance of the sensor—specifically enhancing the sensitivity and specificity towards Myo. The PIP was directly polymerised on the electrode surface and the generated cavities that allowed rebinding of the Myo also provided access to the electrode surface for the redox mediator. This is a fundamental aspect for the mechanism of detection, as by providing access to the electrode surface, this format facilitates redox reactions in the absence of Myo. Hence, the subsequent addition of Myo blocks the electrode and consequentially reduces the electrochemical current relative to the Myo concentration. Figure 5 depicts the PIP synthesis scheme and the detection mechanism, illustrating the reduction in the differential pulse voltammetry peak observed for increasing Myo levels. 

### 3.4. Classic ‘Label-Free’ Techniques

Imprinted biomimetic receptors have also been synthesised for use with classic ‘label-free’ detection techniques. One group have produced a biomimetic sensor that implements surface plasmon resonance (SPR) to detect bound analyte [143]. Targeting cTnT, the authors used epitope and whole protein imprinting, synthesising the polymeric films directly onto a gold-coated surface. This detection mechanism relies on bound analyte initiating a light refractive response at the gold surface that correlates to the changing dielectric environment [144]. In this study, the epitope PIP facilitated the detection of cTnT as low as 518 μg/L, which is slightly below that achieved using the whole protein as the template, albeit far greater than the recommended ‘cut-off’ threshold for the rule-in/rule-out of AMI [145]. Furthermore, the authors implemented an amplification strategy by introducing a secondary antibody for signal enhancement and determined that the performance in diluted serum was comparable to that in buffer solution.

Another detection platform that facilitates analyte measurement independent of labels is the quartz crystal microbalance (QCM). Any mass deposited on the surface of a QCM induces a change in the resonant frequency that is linearly related to the quantity of the adsorbate within specified parameter limits, i.e., area, mass, temperature and is monitored by a counter circuit [146,147]. This detection technique was utilised for the measurement of high-density lipoprotein (HDL)—an inverse CVD risk biomarker in which higher concentrations are indicative of a good cardiovascular outlook—and was captured by synthesised PIPs [148,149,150,151]. Both of the QCM electrodes were coated in a polymeric film to provide an internal reference and eliminate potential discrepancies, with one of the electrodes covered by the NIP. The specificity of the PIP was confirmed in a solution of low-density lipoprotein (LDL), with a considerable decrease in QCM signal observed. An alternative ‘label-free’ detection technique that is frequently employed for analyte measurement in biosensors is electrochemical impedance spectroscopy (EIS). This method involves applying an AC potential frequency sweep to produce a frequency spectrum representative of the amount of bound analyte. The electrical properties at the electrode-solution interface are altered by analyte binding to the immobilised receptor on the electrode surface. One group used EIS as a detection technique for the measurement of myoglobin [152]. Site-directed immobilisation through the formation of amide bonds and subsequent polymerisation was followed by electrochemical measurements that were conducted in a HEPES buffer solution containing potassium ferricyanide as the redox mediator. The authors demonstrated that the biomimetic sensor could only detect Myo down to 2.25 mg/L and that the PIP could distinguish between Myo and selected interfering molecules.

### 3.5. Multiplexed Detection

Multiplexed POCT (xPOCT) incorporates the simultaneous analysis of several biomarkers from a single sample. Detecting multiple cardiac biomarkers offers improved diagnostic and prognostic capabilities that is supported by their inherent physiological function and the differing pathophysiological characteristics they exhibit. Multiplexed POCT improves risk stratification and the prediction of cardiovascular mortality, although its value in diagnosing AMI in contrast to cTn measurements alone is disputed [79,131,153,154,155].

Nonetheless, many biosensors and POC platforms have been developed with multiplexing capabilities, although the same level of progression has not been observed for biomimetic sensing, with few reports of multiplexed sensing strategies and none for the detection of cardiac biomarkers. However, multi-marker analysis has been implemented for some biomimetic sensors, with several groups utilising QDs that were excited by a common wavelength and subsequently emitted distinct wavelengths without any spectral overlap and thus enabling the measurement of individual analytes [156,157,158]. Biomimetic multi-marker detection is not only confined to luminescence-based detection mechanisms, as one group have produced a QCM sensor array for the detection of three analytes [159]. Similarly, three neurological biomarkers were detected simultaneously using MIPs that were dropcast or electropolymerised directly on screen printed electrodes (SPE’s) [160]. Here, the authors evaluate two synthesis approaches, utilising polypyrrole (dropcast) and poly-*o*-phenylenediamine (electropolymerised), with the former being an electrically conductive polymer and the latter an insulating polymer [161,162]. The measurements were obtained by performing differential pulse voltammetry, with multi-analyte detection being supported by the distinct oxidation peaks attributed to each analyte. Furthermore, it appears that simultaneous measurements in a single sample were not explicitly demonstrated, although, the authors conducted cross-reactivity experiments, illustrating the selectivity of the MIPs towards their intended targets.

These multi-analyte detection techniques highlight the adaptability of biomimetic sensing, albeit for the measurement of non-cardiac biomarkers. Moreover, xPOCT is a valuable tool within cardiac diagnostics and one that could be influenced by the development of imprinting techniques in the future.

### 3.6. Feasability for Point-of-Care Testing

**Figure 6:**
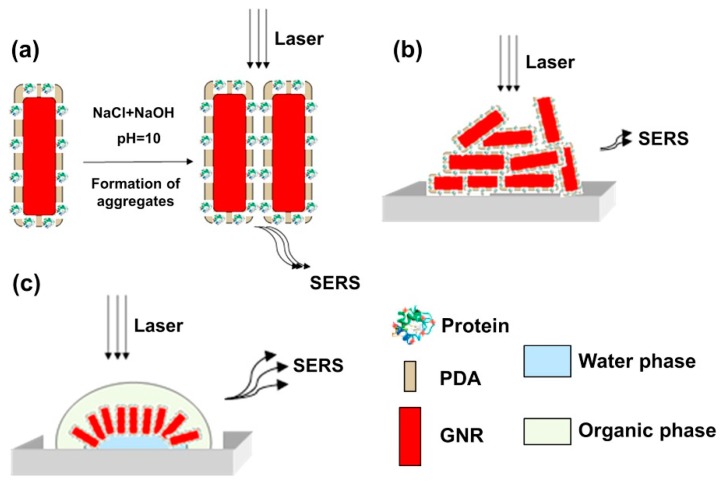
Detection scheme for transferrin measurement using surface-enhanced Raman spectroscopy [163]; SERS—Surface-enhanced Raman spectroscopy, PDA—Polydopamine, GNR—Gold nanorods (Figure was re-printed with permission from the publisher).

Many of the detection techniques that are used in conjunction with PIPs are frequently employed for biosensors, illustrating that although the composition of the receptor may be dissimilar, the use of established detection techniques is feasible. Moreover, the inability to construct sandwich complexes is being addressed by the integration of nanomaterials within the synthesis of PIPs and several alternative approaches have been produced to combat this previous limitation. However crucially, some of the assessed techniques may not be suitable for use in POCT. Besides the lack of sensitivity and the excessive detection times reported for some of the evaluated methods, performing the detection of target analyte using certain techniques requires expensive excitation equipment or complex instrumentation for analysis. This factor is particularly relevant to SPR and also applies to the development of a biomimetic sensor targeting transferrin that utilised surface-enhanced Raman spectroscopy (SERS) as the detection mechanism, which is illustrated in Figure 6 [163]. In this work, the authors have synthesised the PIPs on the surface of gold nanorods, an essential aspect that enhances the Raman scattering effect, and ensured the polymeric film was sufficiently thin (5.3 ± 0.4 nm) to support favorable binding kinetics. The period between protein incubation and conducting the Raman measurements was 20 min, with 4.61 mg/L being the lowest transferrin concentration assessed using this sensing approach, which is considerably less sensitive than some comparable techniques [164,165,166].

**Figure 7:**
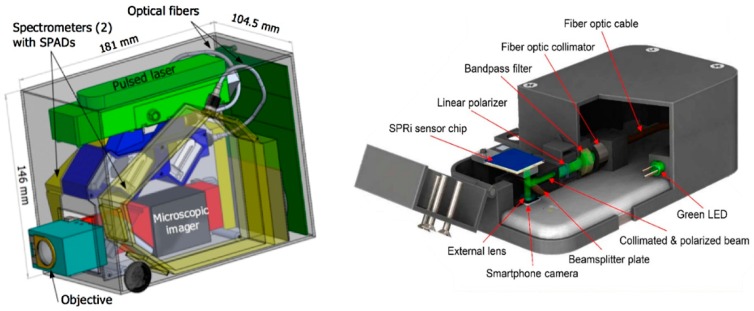
(**Left**) Miniaturised time-resolved Raman instrument [169]; SPAD—Single photon avalanche diode. (**Right**) Portable surface plasmon resonance imaging platform [173]; SPRi—Surface plasmon resonance imaging (reprinted with permission from the publisher).

Additionally, conducting SERS analysis requires a Raman system which is not typically recognised as a low-cost automated instrument [167,168]. However, the progression of related technology, particularly the improvement in diodes for overcoming previously identified issues in bandwidth and sensitivity, has enabled the development of miniaturised and more cost-effective Raman instruments like that shown in Figure 7 [169,170,171]. This is also a prominent concern for SPR, in which a sophisticated instrument is required to measure the shift in refractive index. Although recently, portable miniaturised SPR optical sensor platforms have been reported, with some being compatible for use with mobile phones [172,173,174]. Hence, several of the detection techniques that have been introduced are promising for integration with POCT, reflecting the approaches implemented in the development of cTnI biosensors and POC platforms. Furthermore, despite the challenges encountered concerning the formation of sandwich complexes, the availability of stimuli-responsive polymers and the resourceful manipulation of detectable species enables the optimisation of quenching techniques among others, thus facilitating the accurate detection of cardiac biomarkers.

## 4. Biomimetic Sensors Analytical Performance

Biosensors are central to many commercially available POC platforms, often employing antibodies as the bioreceptor [103]. This preference towards immunoglobulins likely stems from their widespread use in traditional laboratory-based assays, such as enzyme-linked immunosorbent assays (ELISAs) and western blotting. In addition, genetic and protein engineering approaches have allowed the generation of cost-effective recombinant antibodies with improved properties—such as higher affinity for their target analyte—and various structural formats (scFv, Fab, scAb) that are favorable in certain circumstances and applications [175,176,177]. As such, through decades of research and development, antibodies are well characterised and optimised for highly sensitive biomarker quantification. Their popularity in this regard is further supported by the vast number of published studies detailing the development of antibody-based biosensors, many of which target cardiac biomarker detection.

In many instances, biomimetic sensors present a more cost-effective option than that of biosensors, owing to their lower demand on expensive biological reagents and low costs associated with polymer materials. Depending on the protein template used for PIP production, development costs can be cheaper by thousands, with antibodies priced at €100′s per microgram in contrast to €10′s per gram of polymer, in addition to a greatly reduced development period [178,179]. Although, the continuing influence of economies of scale have reduced antibody production costs [180]. Regardless, PIPs offer an attractive option for POC platforms, which frequently feature single-use assay inserts that should be produced at a low cost to ensure clinical feasibility. However, for biomimetic sensors to be considered viable for POCT, they must also demonstrate superior (or at the very least, comparable) analytical performance to biosensors and traditional laboratory assays.

A favourable feature of MIPs is their long-term stability, with some imprinted polymers shown to retain a high level of performance following storage at room temperature for over 2 years [181]. One study has demonstrated the regeneration capacity of MIP-based sensors [182]. In this work, the authors utilised various monomers and cross-linkers, changing the cross-linker/monomer ratio and assessing the performance of the MIPs following 100 regeneration cycles. Certain polymers, such as those consisting of methacrylate and acrylamide cross-linkers degrade after regeneration in acidic conditions, whereas divinylbenzene-based polymers continued to display high affinity after 100 washes. However, divinylbenzene is insoluble in aqueous-based solutions and is rarely employed in the synthesis of PIPs, with acrylamide and methacrylate-based cross-linkers being utilised much more frequently [94]. Other groups have conducted regeneration studies for PIPs targeting cTnI and Myo, with these biomimetic sensors exhibiting little change in affinity towards the target protein after repeated regeneration cycles over several months [183,184,185]. In addition to long-term stability, MIPs can be stable at temperatures above 100 °C and in highly acidic or basic environments without reduced affinity towards the analyte [186,187]. However despite the robust nature of these MIPs, it is unlikely that any POC cartridges would be exposed to these conditions, even in resource-constraint settings.

### 4.1. Evaluating Comparitive Performance

Current commercial cardiac POC platforms are capable of yielding results more rapidly than laboratory-based assays, although this improvement typically comes at the cost of decreased accuracy and sensitivity [188]. In a recent study carried out by Suzuki et al., it was established that the sensitivity of Radiometer’s AQT90 FLEX Analyser was 58.8% when used within the first three h of AMI symptom onset [189]. Similarly, Quidel’s Triage^®^ Cardiac panel displays a clinical sensitivity of just 65.0% during these vital early hours of AMI [190]. Hence, there is considerable scope for improvement within this area, giving biomimetic sensors the opportunity to compete with cardiac biosensors for incorporation within POC-based cardiac diagnostics. Table 2 briefly summarises the testing capabilities of a cross section of recently developed antibody and PIP-based sensors for cardiac biomarker detection.

The LOD and linear ranges presented in Table 2 vary greatly amongst reported sensors—biomimetic and bio-based alike—with LOD values for cTnI sensors alone ranging from 0.025–634.5 ng/L across the two sensor categories. Furthermore, a brief examination of the data presented demonstrates how dramatically the linear range of detection can vary between sensing techniques. Some biomimetic approaches are reported to be capable of quantifying concentrations that span up to 8.43 mg/L [203], while others report much more conservative ranges as narrow as 90 ng/L [196]. Despite the high level of variation, general biomimetic sensor performance has been found to be comparable to that of biosensors, thus exhibiting the ability to detect clinically relevant low levels of targeted analyte.

Due to the extremely low levels of biomarkers that are being examined, an ideal POC device would need to exhibit a very low LOD, with a broad range of detection to provide an accurate assessment of cardiac health. This high level of sensitivity was achieved for the quantification of cTnI, both by biomimetic sensors and biosensors [191,193]. Though in this particular instance, the biosensor developed by Bhatnagar et al. displayed a lower LOD of 0.025 ng/L in comparison with the limit exhibited by the biomimetic sensor of 0.8 ng/L, however, the PIP-based sensor was capable of a much wider detection range. The two sensors performed equally well in terms of response time, both requiring only ten min of an incubation period for sufficient cTnI interaction.

It must be noted that the experimental criteria and methods used to assess the values shown in Table 2 differed amongst studies, naturally leading to disparities between the apparent performances of the various sensors. Perhaps most critical in this regard is the decision of some researchers to report results based on the analysis of spiked buffer samples, spiked serum samples, spiked whole blood samples, or real patient samples, with concentrations and compositions of the fluids varying between studies. These details can often have considerable effects on the resulting LOD and linear ranges observed for these techniques. For example, the cTnI biosensor developed by Singh et al. had a linear response of 0.008–20 μg/L in PBS [205]. However, when tested in serum, a range of 0.8–20 μg/L was observed. In general, the effects of the serum matrix are unclear for biomimetic sensors as the studies that test the performance in serum use heavily diluted serum—up to 1000 times in some instances—and others do not demonstrate the ability to detect the same concentrations reported in buffer solutions [136,140,195]. Notably, the biomimetic sensor developed by Ma et al. was tested in spiked buffer and serum samples, with similarly accurate results being obtained for both [193].

### 4.2. Clinical Considerations

Complex sample matrices such as serum and blood create additional problems regarding the development of sensors for targeted analyte detection. That is, the interaction with, and non-specific adsorption of interfering molecules. In the case of biosensors, various methods have been developed to minimise surface fouling, for example, incorporation of self-assembled monolayers (SAMs) such as poly(ethylene glycol) or 3-mercaptopropionic acid demonstrate anti-fouling properties [206,207,208]. Furthermore, by the nature of their smaller size, recombinant scFv antibodies are known to be immobilised in a more condensed arrangement on the sensor surface. By consequence, this reduces interaction between the sensor surface and any interfering molecules, in addition to the lack of constant regions also reducing non-specific interferences [175]. In a recent study, both antibody immobilisation and prevention of surface fouling were optimised through the employment of synthetic peptides [194]. By producing a novel peptidylated gold surface, oriented antibody immobilisation could be achieved through covalent bonding to the peptides, while simultaneously blocking non-specific adsorption. This study demonstrated the superior sensing performance to that of a SAM-based biosensor, demonstrating the ability to detect cTnI down to a 1.9 ng/L.

Several techniques have also been employed in biomimetic sensors for the minimization of surface fouling, giving PIPs the potential to perform comparatively well in complex serum. Of particular note is the use of phosphorylcholine-immobilised surfaces in the detection of CRP. Phosphorylcholine is the zwitterionic headgroup of phospholipids found on the surface of cellular plasma membranes. It is a known high-affinity ligand of CRP, possessing hydrophilic properties to prevent non-specific protein adsorption [209]. Phosphorylcholine has been proven to be highly effective in the selective capture of CRP from complex biological fluids such as human serum, showing great potential as an alternative to traditional methods [210]. Iwasaki et al. produced a study that utilised Fe_3_O_4_ nanoparticles modified with PC-derived polymer brush structures for the extraction of CRP from simulated bodily fluids [211]. Non-specific protein adsorption was reduced by the presence of these brush structures, and a LOD of 1.25 mg/L for CRP was achieved. This demonstrates the potential for imprinted polymer-based sensing approaches to achieve high selectivity and specificity for their target analytes, with lower susceptibility to biofouling.

High selectivity and biomarker recovery can be further enhanced by the improvement of receptor-analyte binding affinities. The dissociation constant between immobilised antibodies and their target antigens is usually known to vary between 10^−7^ to 10^−11^ M, with some antibodies demonstrating even stronger affinities [212,213]. Beyond the improved capacity to rapidly form stable bonds with the target antigen, receptors with high affinities ensure the antigen will remain bound during the assay and sensor washing steps. Kim et al. developed a PIP for CRP detection that achieved a dissociation constant of 3.3 × 10^−10^ M [109]. This was superior to antibody-based sensors used for comparison at the time of development, which possessed higher dissociation constants of 7.1 × 10^−7^ and 2.6 × 10^−9^ M [214,215]. In a separate study by Karimian et al. a cTnT PIP receptor reported a high affinity for its target biomarker, with an impressive dissociation constant of 2.3 × 10^−13^ M [216].

In a study by Keçili, molecularly imprinted microspheres for selectively detecting Myo in serum samples were designed and characterised [217]. Rapid binding of Myo was observed within the first 40-min of incubation, with a binding capacity of 623 mg/g being recorded, illustrating good agreement between the experimental and the theoretical estimate attained from the Langmuir binding isotherm. The authors appear to imply that the microspheres, possibly due to the increased surface area, facilitate the capture of a higher concentration of Myo, and thus potentially support the fabrication of a biomimetic sensor with a wide linear range of detection. A PIP synthesised to detect cTnI epitopes achieved a maximum binding capacity of approximately 1.5 mg/g within an optimal time of 60 min [218]. In this work, fingerprint analysis was conducted to identify three distinct epitopes that were used as templates for the PIP, which was synthesised in bulk and subsequently grinded to micron dimensions. The detection limit was notable for its high sensitivity at approximately 7.05 ng/L and the approach could perform cTnI enrichment in a complex sample of digested human serum albumin, although conducting the matrix assisted laser desorption ionisation mass spectrometry measurements required an Ultraflex II instrument and other items of equipment. Therefore, this technique is unsuitable POC integration due to the reliance upon sophisticated instrumentation.

Comparison of antibody-based biosensors and biomimetic sensors is further complicated by the necessity to consider the sensor response time. Studies have shown that longer time periods between AMI onset and clinical intervention are associated with an increasingly higher risk of mortality, rising from 3.0% risk at 30 min, to 4.3% after just 90 min [219]. Thus utilising cardiovascular POC platforms has been shown to reduce the testing turnaround time and although many of the sensors reported in Table 2 boast impressive assay times—as low as just one minute—it is worth nothing that these times frequently refer to the incubation of a processed serum sample on the sensor surface only. Sample preparation steps needed to obtain serum from blood are often not included in the response times reported, despite adding considerable delays to the overall ‘sample-to-result’ time. This preparation lag is an issue that is common to both types of sensors, and will therefore have to be addressed and streamlined regardless of which technology is selected for use. Furthermore, evaluating the capability of a sensor or assay to measure cTnI levels in a spiked serum sample does not guarantee the sensing technique will produce a similar response in a clinical setting. In circulation, very little free cTnI is present following cardio myocyte damage, with a large proportion of circulating cTn being cTnT and cTnI-C complex [47].

## 5. MIPs for Point-of-Care Testing

**Figure 8:**
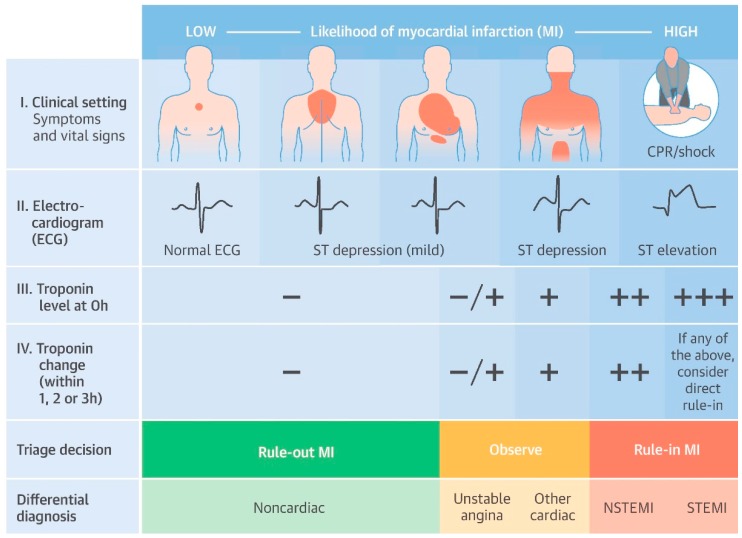
Triage strategy for patients presenting to the emergency department with a suspected acute coronary syndrome [224] (reprinted with permission from the publisher).

To date, the most evident contribution of POCT towards CVD assessment is the diagnosis of acute coronary syndrome (ACS) in acute care settings, despite reported cTn elevations for various other cardiovascular-related illnesses [220,221,222,223]. This typically involves performing cTn measurements from serum samples drawn from patients at presentation to the emergency department displaying symptoms of AMI. Currently, the diagnosis of AMI is dependent on the rise and/or fall of cTn concentrations with at least one measurement above the 99th percentile URL and evidence of one other listed criterion specified by the European Society of Cardiologists (ESC) [11]. An example of a triage strategy to assist the rule-in/rule-out of AMI is presented in Figure 8 [224]. This illustration depicts a typical assessment that a patient presenting to the emergency department suffering from symptoms of an acute coronary syndrome (ACS) may receive. The availability of improved hs-cTn assays has increased the efficacy of AMI diagnosis and has contributed towards the emergence of rapid rule-in/rule-out algorithms, dictated by absolute or differential changes in cTn levels to accelerate the triaging process, in addition to reducing AMI diagnostic costs [16,224,225,226,227,228,229]. Moreover, recent reports support the implementation of POCT for rapid rule-in/rule-out of AMI due to comparable performances to hs-cTnI assays, with one such study demonstrating that the PATHFAST cTnI-II can be considered a hs-cTnI assay [15,18]. These highly impressive characteristics are not observed in the majority of POC platforms for cTn measurements, with significant cTn measurement discordance between POC platforms in addition to the prevalence of false positive and false negative readings [46,103,230]. The challenge for MIPs in influencing the development of POC platforms for cTn measurements is that common features, such as their low cost, rapid synthesis and long-term stability become extraneous in acute settings where sensitivity and a rapid response are imperative.

### Cardiovascular Risk Stratification

**Figure 9:**
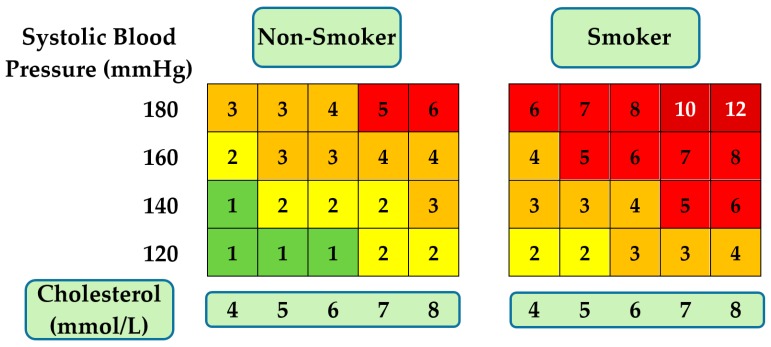
Relative risk chart derived from SCORE risk estimation system—Risk indicator values outline the 10 year risk of developing CVD.

The provision of cardiovascular screening services is an essential measure for the prevention of CVD. These routines include biomarker analysis to evaluate the risk of developing CVD or to identify its early onset and are most often conducted in primary care settings. Moreover, a high proportion of deaths attributed to CVD occur in asymptomatic individuals who may have been classified as high-risk following a cardiovascular screening [231,232,233,234]. Several cardiovascular risk estimation systems have been developed to assist in the risk profiling of patients, with Figure 9 depicting an adapted and simplified SCORE risk estimation chart that displays only a limited selection of key risk factors [235,236]. One group have developed biomimetic sensors for the selective detection of high-density (HDL) and low-density (LDL) lipoproteins in serum [237,238]. These are vital biomarkers for cardiovascular risk prediction as they directly affect the build-up of atherosclerotic plaque and are assessed in conjunction with triglycerides to provide a lipid profile for standard cardiovascular screening procedures [239,240,241,242,243,244].

Additionally, there is a reported absence of cholesterol testing in 40% of global public health sectors and though some studies have demonstrated an overall decrease in total serum cholesterol [245,246], including LDL, some more recent reports indicate an increasing trend in bad cholesterol (LDL) serum levels [247,248,249]. Crucially, a reduction in cholesterol levels is associated with attending cardiovascular screenings and thus illustrates the beneficial impact from raising awareness on cardiovascular health. In relation to the cholesterol biomimetic sensors, the synthesised PIPs facilitated the detection of clinically relevant levels of HDL and LDL in diluted serum and displayed similar performance characteristics to reported biosensors. In particular, the HDL sensor could complete measurements within 10 min and had a coefficient of variation below 10%, a similar variability to high-sensitivity assays. These levels of performances ensure that the lipoprotein biomimetic sensors are potential candidates for POC integration.

**Figure 10:**
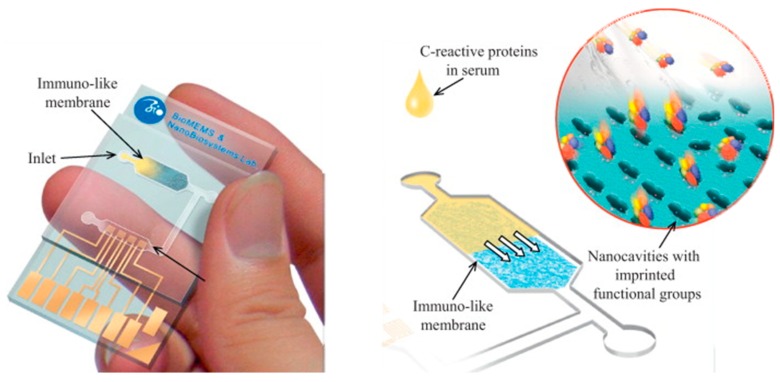
Microfluidic biochip containing polymer membrane for CRP detection [255] (reprinted with permission from the publisher).

Another biomarker often measured to improve the accuracy of the risk derived from cardiovascular screening is CRP [250,251,252,253,254]. Several biomimetic sensors have been developed for the detection of CRP, exploiting the affinity of this inflammatory protein towards its natural ligand and thus synthesising PIPs from PC analogues. This approach has been adopted for the development of a microfluidic biochip targeting the rapid detection of CRP [255]. Utilising O-(4-nitro-phenylphosphoryl) choline as the functional monomer, the authors demonstrated the improved sensing performance attributed to oriented immobilisation of the template on the gold surface prior to polymerisation. Although, unlike conventional protein templates, this complex template comprised of anti-CRP antibody and captured CRP. Seemingly, the authors intended to improve the ordered assembly of the template by utilising the complex template, which is possibly superior to the template protein alone, as the oriented antibodies will consistently bind to CRP at a specific epitope. However, functionalisation of the gold surface with cysteamine and glutaraldehyde to achieve amine-coupling will not ensure uniformly oriented antibodies, thus the extent of specified orientation is limited [256]. The polymer membrane was subsequently incorporated within a plastic microfluidic chip that contained an enclosed interdigitated electrode array, as shown in Figure 10. The detection technique was based upon the rate of decay of the applied electric signal due to impedance changes that correlated to the CRP content of the test sample. Thus, rapid detection of CRP was achieved in less than 2 min after initial incubation with spiked serum samples. However, the sensor was shown to detect CRP down to 10 mg/L, a level that exceeds the low to high-risk categories recommended and therefore only facilitates the measurement of very high-risk categories [253,254]. Nonetheless, it is apparent from the dynamic response of the decaying signal that this approach has the potential to detect lower CRP concentrations and provide the basis for an improved clinically applicable biochip for POCT.

Cardiac risk stratification is an essential process for profiling high-risk patients and one that could benefit considerably from the emergence of adequate biomimetic sensors for the detection of relevant biomarkers. Hence, these sensors could become fundamental elements of low-cost POC devices within standard cardiovascular screening procedures and support further expansion of cholesterol testing globally. Point-of-care testing has been shown to improve the efficacy of cardiovascular risk interventions by improving the efficiency of the process and facilitating rapid biomarker analysis prior to a formal consultation with a primary care provider [257]. The implementation of POCT of cholesterol levels in pharmacies has been raised and evaluated as a service to precede primary care intervention [258]. Several studies have conducted POCT of lipids in pharmacies and in general, have confirmed similar accuracy to laboratory testing, although, POCT of HDL produced inaccurate measurements [259,260,261]. Notably, some of the cartridges used required refrigeration, potentially restricting the application in resource-constraint settings. Hence, this is an area of cardiovascular diagnostics that could benefit from the emergence of a more robust, cost-effective device to support increased accessibility to cardiovascular screenings. Furthermore, the widespread availability of less costly POC devices for cholesterol testing, with increased shelf-life, could impact the provision of screening services, potentially shifting some responsibility from primary care.

## 6. Conclusions

The development of POC platforms has traditionally involved the incorporation of biorecognition elements to capture and separate analyte from a sample matrix. These platforms have become increasingly important in acute care settings, facilitating the rapid analysis of cardiac biomarkers—chiefly the cTns—and have reduced the length of stay for patients in emergency departments. Moreover, there is a particular focus on improving the sensitivity of POCT methods to emulate the performance of central laboratory assays, with some of the latest platforms demonstrating comparable analytical characteristics. However, it is essential that POC platforms adhere to recommended standards that support rapid sample analysis in a patient’s proximity independent of sophisticated instrumentation. Furthermore, several challenges complicate the accurate detection of cTn, such as the presence of skeletal troponin isoforms and interfering molecules, a lack of standardisation and the differing 99th percentile URL between genders.

Molecularly imprinted polymers can be employed as biomimetic receptors to capture target analyte, similarly to biorecognition elements. These PIPs have their own inherent challenges that may compromise the ability to selectively bind to cardiac biomarkers in a complex sample. Issues regarding cavity homogeneity and template extraction can limit the synthesis of high affinity biomimetic receptors. Additionally, limited solvent selection and unfavorable binding kinetics may deter the adoption of these receptors for developing POC platforms. Furthermore, synthesising PIPs using cTnI as the template may not produce an effective receptor for real sample analysis as cTnI is primarily bound to troponin C in circulation. These challenges and performance limitations may somewhat restrict the application of imprinting technology for diagnostic platforms, however, numerous enhancement methods can mitigate the negative influence for the selective capture of analyte. Enzymatic digestion in conjunction with washing the PIP with a surfactant can increase the availability of high affinity binding sites, whereas utilising dummy templates has been shown to reduce template leaching during analyte detection. Additionally, alternative synthesis techniques, such as epitope imprinting, enables polymerisation in solvents that may typically disrupt the conformational structure of whole proteins and facilitates the use of otherwise incompatible functional monomers. The ability to synthesise PIPs from ligand analogues and the natural affinity of boronic acids towards glycans also enables the synthesis of PIPs that can exploit inherent properties of target molecules. Moreover, the performance of some biomimetic sensing techniques reflects that of reported biosensors, utilising classic transducer mechanisms in addition to single step detection techniques and crucially, several of these sensors have demonstrated rapid sample analysis, although primarily in spiked buffer. Hence, these sensors—which display superior stability, lower production costs and comparative performances to biosensors—can be viewed as promising alternatives for the development of future POC devices.

However, the incorporation of PIPs within medical diagnostic platforms requires extensive assessment and trials in clinical settings. The display of high sensitivity in a laboratory environment using spiked solutions does not necessarily imply that practical challenges encountered in standard routines and in a complex matrix, like serum, can be easily overcome. These factors suggest that adapting this technology for cardiovascular biomarker assessments in time-critical acute care settings is currently unfeasible. Although, cardiovascular diagnostics span beyond the domain of AMI diagnosis and encompass a variety of test procedures that are frequently implemented in contrasting settings. Risk stratification is a critical element of CVD prevention and one that POCT has been shown to positively influence. Moreover, current POCT of lipid panels has demonstrated measurement deficiencies in comparison to laboratory testing, with some devices requiring refrigeration. This highlights the need for further development and may eventually transpire to be an opportunity for PIPs to contribute to improved performances, emerging as a cost-effective alternative to biorecognition elements and potentially increasing the prevalence of POCT for use in cardiovascular risk screening in rural and resource-limited settings.

## Figures and Tables

**Table 1:** Challenges of protein imprinted polymer synthesis and the significance for sensor performance.

Challenges of PIP Synthesis	Potential PIP Deficiencies		Significance for Sensor Performance
	Description	Illustrations	
Template/ Monomer/ Cross-linker Ratio	Excessive cross-linker can block access to binding sites, therefore restricting mass transfer	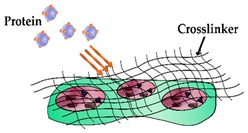	The time period required to perform the measurements is increased
	Suboptimal mixture can result in a PIP with a lack of binding sites	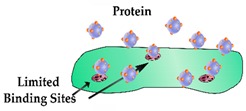	Limited detection range
	A low cross-linker ratio can lead to binding sites lacking rigidity	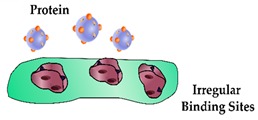	Recognition element with low specificity and an increase in non-specific adsorption
	Excessive cross-linker can lead to template entrapment	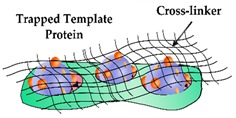	Inability to rebind target analyte due to binding sites being occupied/ inaccessible
Monomer/ Cross-linker/ Solvent Selection	Solvent selection is typically limited to aqueous-based to ensure proteins retain conformational structure	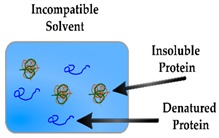	Possible production of imprinted polymers with poor performance characteristics
	Monomer and cross-linker selection restricted to those soluble in aqueous-based solvents	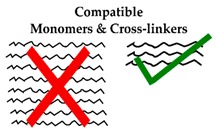	
Template Extraction	Harsh extraction solvents can damage binding sites and reduce binding site homogeneity	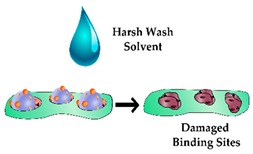	Heterogeneous binding sites have poor specificity and can also affect binding kinetics
	Incomplete template removal can produce template leaching during measurements	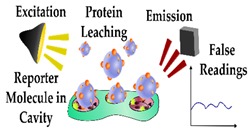	Release of template protein while performing measurements can induce a false response
	Suboptimal extraction technique can result in high affinity binding sites retaining the template protein	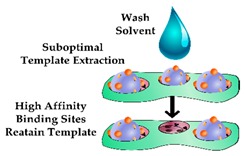	Only low affinity binding sites remain accessible, thus, the PIP will have low specificity towards the template
	Enzymatic digestion can partially remove the template protein from binding sites	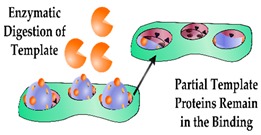	Occupancy of binding sites can inhibit rebinding and therefore limit the detection range

**Table 2:** Analytical performance of assays, biosensors and biomimetic sensors for cardiac biomarkers.

Sensor Type	Target Biomarker	LOD (ng/L)	Linear Range (ng/mL)	Response Time * (min)	Reference
Biosensor	cTnI	0.025	0.000166–16.5	10	[191]
Biomimetic	cTnI	0.5	0.01–5.00	ND	[185]
Biosensor	cTnI	0.7	0.001–100	1	[192]
Biomimetic	cTnI	0.8	0.005–60	10	[193]
Biosensor	cTnI	1.9	0.00001–1.0	30	[194]
Biomimetic	cTnI	634.5	1.18–117.5	5	[195]
Biomimetic	cTnT	6	0.01–0.1	30	[196]
Biosensor	cTnT	7	0.005–0.065	40	[197]
Biomimetic	cTnT	9	0.009–0.8	10	[198]
Biosensor	cTnT	33	0.1–10	60	[199]
Biomimetic	MYO	0.045	0.000304–0.571	30	[136]
Biosensor	MYO	0.4	0.001–100	130	[200]
Biosensor	MYO	10	0.01–100	10	[201]
Biomimetic	MYO	26300	100–1000	25	[184]
Biosensor	CRP	5.0	0.01–1000	60	[202]
Biomimetic	CRP	500	180–8510	3	[203]
Biosensor	CRP	22000	62.5–6250	30	[204]

* Response Time—This includes the incubation period and the time period to acquire measurements, ND—Not disclosed.
